# Methodologies to evaluate the effectiveness of knowledge translation interventions

**DOI:** 10.1186/1710-1492-6-S4-A11

**Published:** 2010-12-10

**Authors:** Monika Kastner

**Affiliations:** 1 Department of Health Policy Management and Evaluation, Faculty of Medicine, University of Toronto, Toronto, ON, M5S 1A1, Canada

## 

Knowledge translation (KT) promotes evidenced-based medicine but methods used to support related practices are often not evidence based [1,2]. Implementation research is complex, as it requires taking into account multiple levels (from patients, multidisciplinary healthcare teams and health facilities to local and national health care systems), which adds to the significant conceptual and methodological challenges that currently exist. These challenges are likely the reasons why the impact of implementation strategies has been modest and why conclusions that can be drawn from these approaches and how they should be applied to given settings are so limited.

There is pressure to improve quality of care, but there is a lack of information about which interventions work and under what circumstances. Most studies registered in the Cochrane collaboration are RCTs (~350,000), but only 2,400 are experimental and quasi-experimental trials of interventions to improve health care delivery. There is a need to shift the focus from developing new treatments to developing approaches that deliver what is already known to work and to create and evaluate interventions from evidence-based knowledge. Given the limited evidence base to work from, people involved in quality improvement (QI) have a responsibility to evaluate the effectiveness of their efforts not only because many interventions are ineffective and may lead to a waste of resources, but also because evaluation creates knowledge that may benefit others.

When considering how to evaluate the impact of an intervention, one should first consider whether the interest is in local knowledge (*i.e.,* whether an intervention worked in the context in which it was implemented, which is of interest to managers responsible for QI within an institution) or generalizable knowledge (*i.e.,* whether an intervention is likely to work in comparable settings which is of interest to KT researchers). Determining the need for local or generalizable knowledge and available resources drives most choices in KT intervention study designs. Evaluation study designs include randomized controlled trials (the gold standard for assessing causality and impact of interventions); and non-randomized or quasi-experimental designs (*e.g.,* controlled/uncontrolled before-after, and interrupted time series designs), which are more subject to biases but require fewer resources. These designs vary in their ability to control for bias to increase internal validity, but even the perfectly valid study may not determine the degree to which the results can be generalized to real practice conditions. Pragmatic study designs can facilitate this by maximizing the relevance of the results for real world decision-making, often for a broad range of settings. Despite a large number of studies, many knowledge gaps still remain. Rigorous evaluation of QI initiatives (using both quantitative studies to better understand “if” something works supplemented by qualitative studies to understand “why”) are needed to increase our knowledge of KT and to improve quality of care.
